# Retinal vessel tortuosity and fractal dimension in diabetic retinopathy

**DOI:** 10.1186/s40942-025-00688-z

**Published:** 2025-06-12

**Authors:** Fariztah Sukainah Nur Fathimah, Sauli Ari Widjaja, Wimbo Sasono, Ima Yustiarini, Muhammad Firmansjah, Ady Dwi Prakosa, Aulia Kezia Mulyazhara, Soebagijo Adi Soelistijo

**Affiliations:** 1 Department of Ophthalmology, Faculty of Medicine, Universitas Airlangga, Surabaya, East Java Indonesia; 2 Department of Ophthalmology, Dr. Soetomo General Academic Hospital, Surabaya, East Java Indonesia; 3 Department of Internal Medicine, Dr. Soetomo General Academic Hospital, Surabaya, East Java Indonesia

**Keywords:** Retinal tortuosity, Fractal dimension, Diabetic retinopathy, Diabetes mellitus, Vascular geometry, Good health and well-being

## Abstract

**Background:**

Retinal vessel geometry characteristic have been studied as one of the signs of microvascular changes in diabetic retinopathy (DR) that necessitates early screening. This study aimed to investigate the differences in retinal vessel tortuosity (VT) and fractal dimension (FD) between patients with and without DR.

**Methods:**

This retrospective study analyzed medical records and OCT-A images of DR and No-DR patients. DR severity was graded by a vitreoretinal specialist following the International Clinical Diabetic Retinopathy and Diabetic Macular Edema Severity Scales. Retinal VT and FD were quantified using ImageJ software. Comparison between groups using non-parametric and Generalized Estimating Equations (GEE) statistical analysis combined with cluster bootstrapping.

**Results:**

We analyzed 96 (161 eyes) with the mean age of 52.7 ± 9.9 years. Compared to No-DR, VT was significantly higher in all DR groups (*p* < 0.05). Mild non proliferative DR (β = +0.0621), Moderate NPDR (β = +0.0412), Severe NPDR (β = +0.0441), and proliferative DR (β = +0.0404). FD of the superficial capillary plexus (SCP) showed no significant difference among the groups and a significantly lower FD of the deep capillary plexus (DCP) compared to the No-DR groups (moderate NPDR (β = -0.0131), severe NPDR ( β = -0.0316) and PDR ( β = -0.0326)).

**Conclusion:**

Compared to No-DR group, VT was found significantly higher in DR group, and FD of the DCP found significantly lower in the DR group. These parameters offer unique insights beyond simple vessel loss and complementary information into the geometric complexity and structural alterations of the retinal microvasculature in DR.

## Introduction

Diabetes mellitus (DM) is a metabolic disorder marked by hyperglycemia, resulting from abnormalities in the production or function of insulin. DM is a prevalent health issue on a global scale, impacting 463 million individuals between the ages of 20 and 79. The International Diabetes Federation predicts that this number will rise to 700 million by the year 2045. Diabetic retinopathy (DR) is a common microvascular condition that frequently leads to blindness in both industrialized and poor countries. The current method of screening for DR involves visually examining fundus pictures to clinically assess the presence of DR. However, there is an urgent requirement for a screening method that can detect DR at an early stage, before it becomes clinically apparent [1–4]. 

Fundus fluorescein angiography (FFA) has a fairly high sensitivity in detecting early changes related to DR such as microaneurysms and retinal capillary permeability, but is not clinically indicated for early detection of nonproliferative DR (NPDR) due to its side effects. Examination with optical coherence tomography-angiography (OCT-A) provided depth-resolved in vivo observation of the retinal and choroidal microvasculature quickly, non-invasively, and able to detect vascular changes in the eyes of DM patients even without clinical DR [5–7]. 

The characteristics of retinal vessel geometry, such as retinal vessel diameter and density, vessel tortuosity (VT), fractal dimension (FD), branching angle, and length to diameter ratio, have been studied as one of the signs of microvascular changes in DM, and are related to its complications including diabetic nephropathy and cardiovascular disease. However, several studies that measure retinal vascular geometry in DM patients with different methods and software have shown inconsistent results [3, 5, 8–10]. 

Retinal VT is one of the early signs of vascular changes in retinopathy cases. Blood vessel tortuosity is known to occur due to hemodynamic changes that cause mechanical instability of blood vessels, endothelial dysfunction, and changes in blood flow in the remodeling process. Studies have shown that there is an increase in retinal arteriole tortuosity associated with mild and moderate NPDR. Another study showed that increased retinal VT is closely related to vascular abnormalities and degenerative processes compared to DR. Another retinal geometry parameter that is starting to be widely studied is FD, which provides a global index to quantitatively assess the geometric complexity of the retinal vascular network, including all branching patterns. This could be an effective indicator of microvascular disease earlier before there are clinical signs of retinopathy. Several studies have found that low FD values ​​indicate less frequent blood vessel branching patterns associated with the risk of cardiovascular mortality and advanced stages of DR [5, 6, 11]. 

Building upon our previous findings that elevated neutrophil–lymphocyte ratio (NLR) is significantly associated with increased arterial stiffness in type 2 DM patients [12]this study shifted focus on the retinal VT and FD using OCT-A images. We also confirmed the close association between renal impairment, DR, arterial stiffness, and retinal neurovascular parameters [11]. Given that vascular complications in diabetes affect both macrovascular and microvascular structures, altered retinal VT and FD may reflect early microvascular changes linked to inflammation or arterial stiffness, serving as potential indicators of broader cardiovascular risks. By utilizing OCT-A and analyzing parameters such as VT and FD, this study aimed to identify subtle microvascular changes that precede clinical DR. Additionally, it seeks to validate findings from our previous studies within the specific population in Indonesia, contributing to a more localized understanding of DR progression and enhancing the potential for personalized screening protocols in this setting.

## Methods

### Study design and population

This retrospective observational study was a part of the Diabetic Ocular Renal Surabaya (DiORS) Study conducted from August 2019 to January 2020 [12]. The study protocol was approved and exempted by the Institutional Review Board of the Dr. Soetomo General Academic Hospital (Komisi Etik Peneliti Kesehatan Rumah Sakit Dr. Soetomo 1551/LOE/301.4.2/XII/2023) and no informed consent needed.

The study used OCT-A scan images and medical record data from participants diagnosed with diabetes mellitus that joined DiORS Study with complete scan of OCT-A. ImageJ software and Fractalyse tools were used to quantify VT and FD from OCT-A scan images. The excluded patients were patients with poor quality OCT-A (signal strength < 7/10). The total samples through purposive sampling were 96 patients (161 eyes), which were divided into 5 groups. DR severity was graded based on the fundus examination findings documented in the medical records by vitreoretinal specialist. The severity of DR were graded according to The International Clinical Diabetic Retinopathy and Diabetic Macular Edema Disease Severity Scales and classified as No-DR, Mild Non Proliferative DR (NPDR), Moderate NPDR, Severe NPDR and Proliferative DR (PDR) [13]. 

### Collection of demographic, clinical characteristic, and laboratory data

Demographic and clinical characteristics, including age, gender, haemoglobin A1c (HbA1c), best corrected visual acuity (BCVA) calculated using ETDRS chart with LogMAR conversion, retinal VT and FD were obtained.

### Measurement of retinal vessel tortuosity (VT) and fractal dimension (FD)

Data from OCT-A exams were collected. Using a 6 × 6 mm scan (perifovea under pupil dilation, macular angiography imaging was obtained using the Cirrus HD-OCT 5000 with AngioPlex software (Carl Zeiss Meditec, Dublin, California, USA; V.10.0). Following segmentation of the en face OCT-A image, the superficial capillary plexus (SCP) and deep capillary plexus (DCP) images were analysed using ImageJ software version 1.53t along with the plugins used for vessel segmentation and length measurement.

**Fig. 1:**
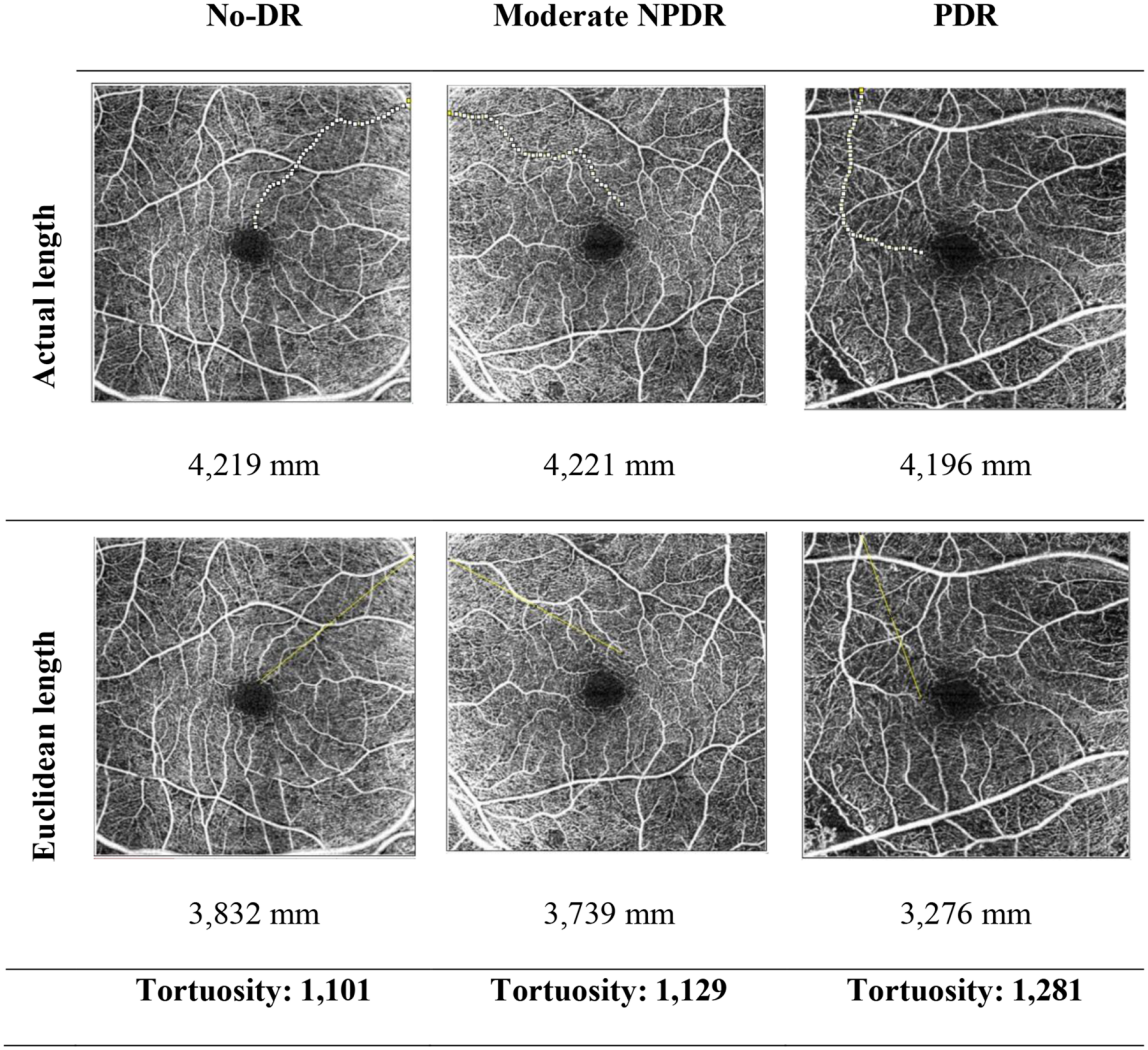
Representative image of tortuosity measurement from superficial capillary plexus OCT-A scan image. No DR, nondiabetic retinopathy; NPDR, non-proliferative diabetic retinopathy; PDR, proliferative diabetic retinopathy; OCT-A, ocular computed tomography-angiography

**Fig. 2:**
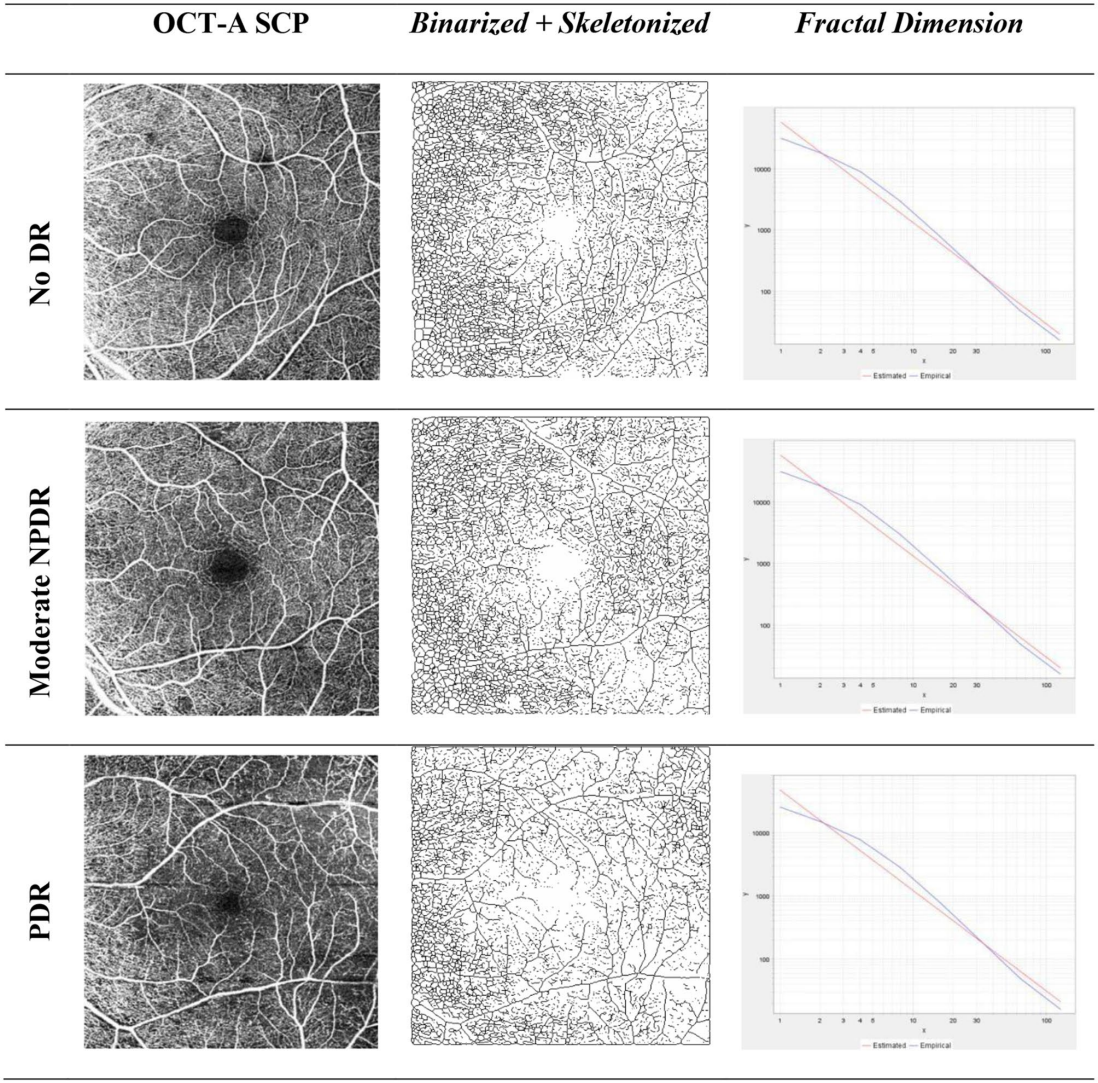
Representative image of the fractal dimension measurement process in the superficial capillary plexus of the No DR, Mild NPDR and PDR groups. No DR, nondiabetic retinopathy; NPDR, non-proliferative diabetic retinopathy; PDR, proliferative diabetic retinopathy; SCP, superficial capillary plexus; OCT-A, ocular computed tomography-angiography

**Fig. 3:**
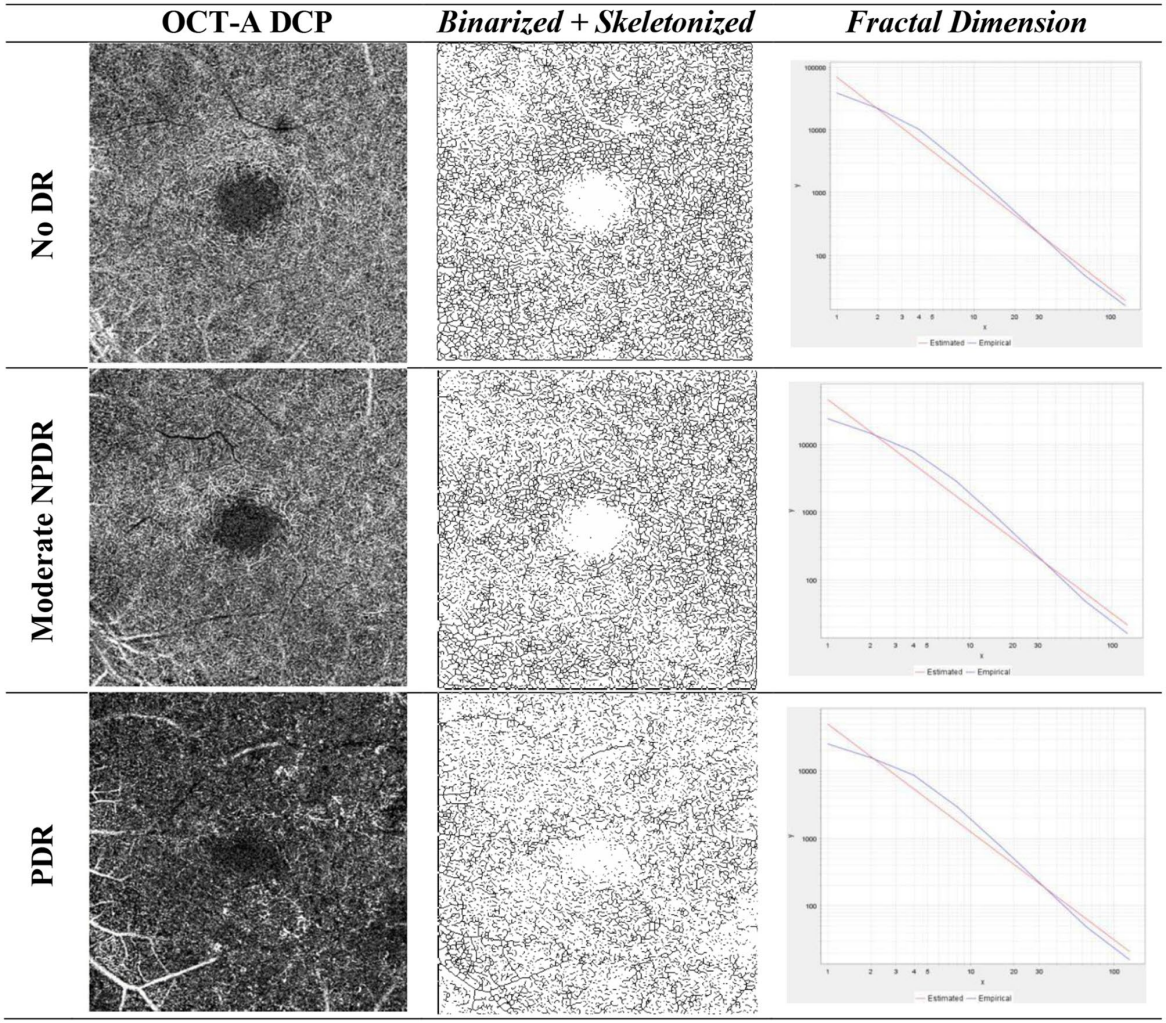
Representative image of the fractal dimension measurement process in the deep capillary plexus of the No DR, Mild NPDR and PDR groups

The calibration of ImageJ using the scale bar embedded in the OCT-A images, enabling conversion from pixels to millimeters for accurate Euclidean distance measurement. The scanned images from Zeiss OCT-A machine gave us the size of the area of macula scanned, which captured 6 × 6 mm of the macula. Then using the information of pixels of the scanned images, we adjusted how many pixels corresponds to the size of the images in millimeters using the menu analyze, and then set scale option. There will be distance in pixels (e.g. 300 pixels/mm) and known distance (e.g. 6 mm). Retinal VT measurement using the skeletonize plugin was performed by taking SCP images and then importing them into ImageJ software (Fig. 1). Quantification of the actual blood vessel length and the Euclidean distance (straight line) from the starting point to the ending point on the OCT-A (en face) image performed by one trained ophthalmologist who were masked to the DR status of the eyes. The ratio between the actual length and the Euclidean distance was calculated using Microsoft Excel. The result was the comparison value of the blood vessel length with the Euclidean distance on the SCP [14, 15]. While retinal FD was measured by taking binarized OCT-A (en face) images of SCP (Fig .2) and DCP (Fig. 3), skeletonized plugin, analyzed using fractalyse software version 3.0 and the default parameters using the box-counting method. The result is the average value of the logarithmic graph of the SCP and DCP complexity patterns [16–18] (See Figs. 1, 2 and 3.

### Statistical analysis

Data analysis in this study consisted of descriptive and inferential analysis conducted using Generalized Estimating Equations (GEE) analysis. Stata version 17 (StataCorp, College Station, TX, USA) was used to perform all the statistical analyses. Descriptive analysis of ratio data was presented in tabulation form with mean ± standard deviation, while categorical data was presented in the form of frequency distribution tables. Then, the differences among 5 groups (No-DR, Mild NPDR, Moderate NPDR, Severe NPDR and PDR) were tested using Kruskal-Wallis and Chi-Square tests. Given that some patients contributed both eyes, all statistical analyses were performed using inferential analysis with GEE to account for potential intra-subject correlation, with patient ID as the clustering variable and an exchangeable correlation structure.

**Fig. 4:**
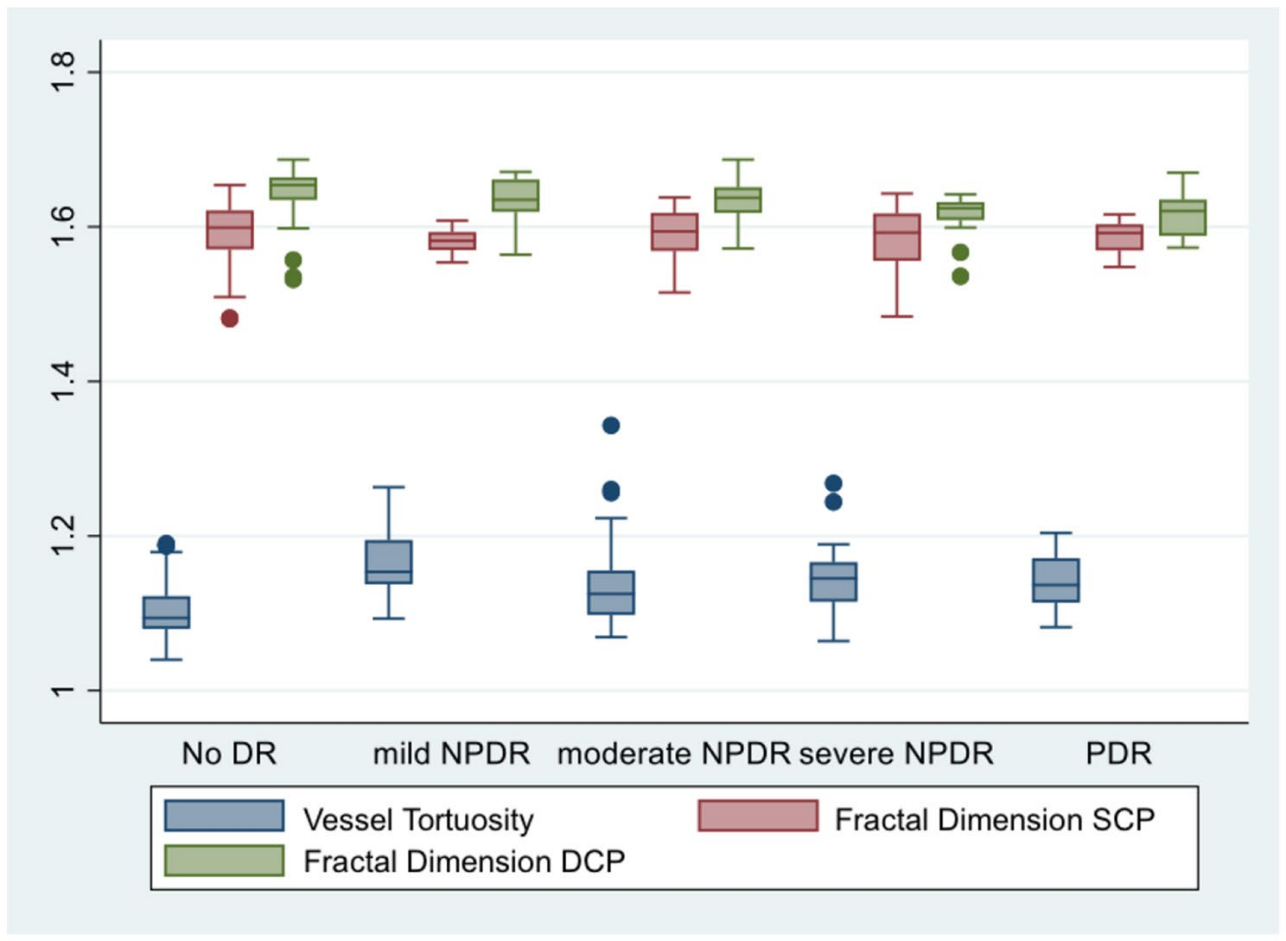
Box plots of mean distribution of blood vessel tortuosity and fractal dimension in the superficial capillary plexus and deep capillary plexus

Before performing data analysis, data distribution testing between groups was carried out. The results of the data distribution test with boxplots showed that the distribution of VT and FD data, for both SCP and DCP, between groups tended to be different (Fig. 4). In addition, the sample sizes between groups also differed. Therefore, GEE analysis combined with bootstrapping was used. This approach is able to obtain more accurate confidence intervals, overcome classical distribution assumptions that may not be fully met, especially if the number of subjects is small, and can provide more robust variance estimation for intra-subject correlation when both eyes were taken into account. Bootstrapping (especially cluster bootstrapping, which is resampling based on individuals, not eyes) provides a more robust variance estimation for intra-subject correlation. Statistical values were declared significant if *p* < 0.05.

## Results

**Fig. 5:**
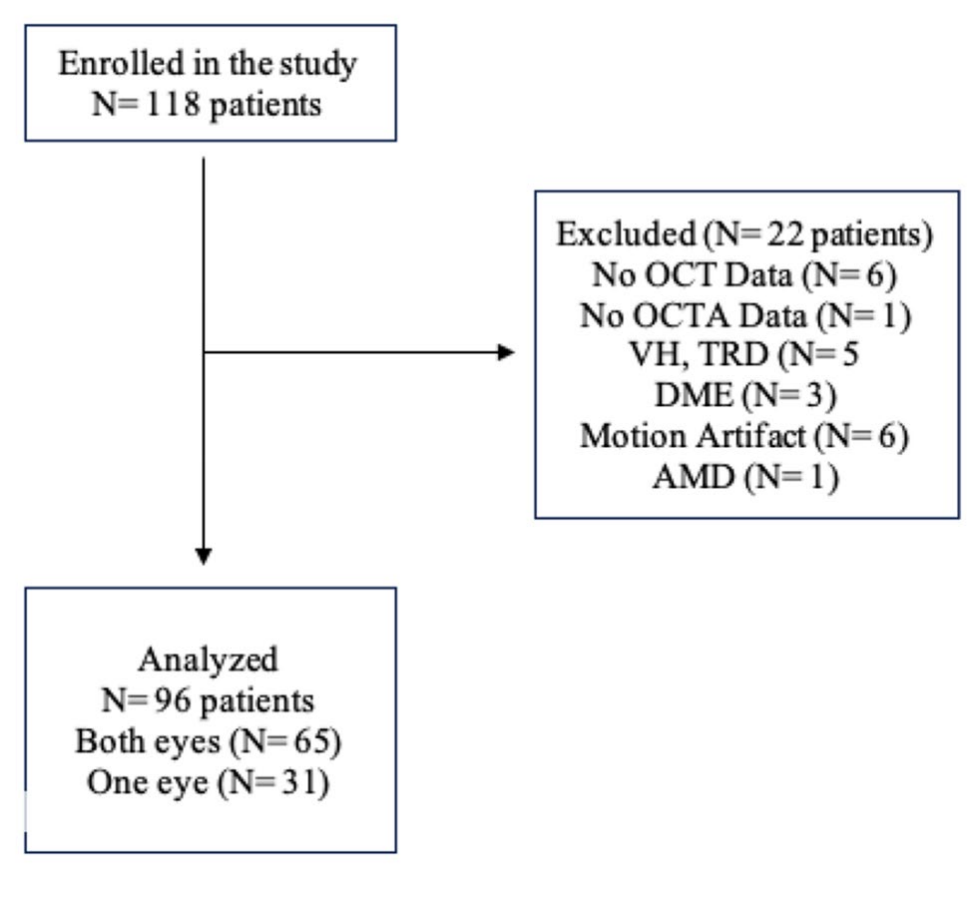
Flowchart of patient enrollment

A total of 118 patients were included in this study, however, 22 patients were excluded due to incomplete medical records (incomplete data of OCT/OCT-A imaging), the presence of other retinal disease (vitreous hemorrhage, tractional retinal detachment, macular degeneration, diabetic macular edema) and motion artefact. From 65 patients, with both eyes (130 eyes) and 31 patients with only one eye (31 eyes) were taken into account leaving 161 eyes were included in the final analysis (Fig. 5).

A total of 96 patients (161 eyes) with the age ranging from 18 to 76 (52.7 ± 9.9) years. Among them, 85 (52.7%) eyes were in the No-DR group, 16 (0.09%) had mild NPDR, 30 (18.6%) had moderate NPDR, and 18 (11.1%) had severe NPDR, and 12 (0.07%) were classified as PDR. In the PDR group, most respondents were male, whereas in other groups, most respondents were female. The HbA1c values in the No-DR, Mild NPDR, Moderate NPDR, and Severe NPDR groups tended to be similar, above 8%, but in the PDR group yielded HbA1c above 10%. Regarding BCVA values, the highest average was shown in the PDR group with an average of LogMAR 0.26, and the lowest was found in the No-DR and Moderate NPDR groups with the average of LogMAR 0.02 and 0.0, respectively (Table 1).

**Table 1:** Demographic and clinical characteristics based on diabetic retinopathy classification

Variable	No DR (*n* = 85)	Mild NPDR (*n* = 16)	Moderate NPDR (*n* = 30)	Severe NPDR (*n* = 18)	PDR (*n* = 12)	*P*-value
Mean age, years	53,1 ± 11,4	53,6 ± 10,8	52,9 ± 7,8	51,2 ± 6,0	51,1 ± 7,0	0,601*
Sex, Male	37 (43,5%)	7 (43,7%)	14 (46,7%)	5 (27,8%)	7 (58,3%)	0,557**
Sex, Female	48 (56,5%)	9 (56,3%)	16 (53,3%)	13 (72,2%)	5 (41,7%)	
HbA1c (%)	8,1 ± 1,8	8,5 ± 1,8	8,8 ± 2,2	8,2 ± 2,2	10,2 ± 3,1	0,105*
BCVA (LogMAR)	0,02 ± 0,06	0,16 ± 0,38	0,05 ± 0,10	0,10 ± 0,12	0,26 ± 0,52	0,002*

* Kruskal Wallis, ** Chi Square

Based on the results of difference tests using Kruskal-Wallis and Chi-Square, it was found that only BCVA yielded a significant difference among the groups. While age, gender, and HbA1c showed no significant differences among the groups (Table 1).

### Differences in retinal vessel tortuosity based on diabetic retinopathy severity

Based on the results of GEE analysis with bootstrapping, it was found that the Wald Test value generated in the mean VT model yielded a p-value < 0.05. This indicated a significant difference in mean VT among the groups. A significant difference in the mean VT between the mild NPDR, moderate NPDR, severe NPDR, and PDR groups compared to the No-DR group was found. The coefficient value for mild NPDR was positive, at 0.0621, indicating that the mean VT value of the mild NPDR group was 0.0621 higher compared to No-DR. The coefficient value for moderate NPDR was positive, at 0.0412, indicating that the Mean VT value of the moderate NPDR group was 0.0412 higher compared to No DR. The coefficient value for severe NPDR was positive, at 0.0441, indicating that the Mean VT value of the severe NPDR group was 0.0441 higher compared to No DR. Finally, the coefficient value for PDR was positive, at 0.0404, indicating that the Mean VT value of the PDR group was 0.0404 higher compared to No DR (Table 2).

**Table 2:** The differences in retinal vessel tortuosity and fractal dimension **Based on diabetic retinopathy classification**

Variabel	Vessel Tortuosity	Fractal Dimension SCP	Fractal Dimension DCP
**Diagnosis**			
Mild NPDR	0.0621	-0.0084	-0.0134
	(0.0121)	(0.0059)	(0.0084)
	0.000*	0.156*	0.111*
Moderate NPDR	0.0412	-0.0019	-0.0131
	(0.0121)	(0.0073)	(0.0059)
	0.001*	0.789*	0.026*
Severe NPDR	0.0441	-0.0083	-0.0316
	(0.0121)	(0.0104)	(0.0070)
	0.000*	0.424*	0.000*
PDR	0.0404	-0.0065	-0.0326
	(0.0122)	(0.0073)	(0.0087)
	0.001*	0.376*	0.000*
**Eye**			
OS	-0.010	-0.0108	-0.0075
	(0.007)	(0.0057)	(0.0044)
	0.156*	0.058*	0.089*
**_cons**	1.105889	1.5968	1.6511
	(0.0051)	(0.0048)	(0.0035)
	0.000*	0.000*	0.000*
**Wald Test**	50.81 0.0000*	6.55 0.2562*	33.77 0.0000*

No-DR, no diabetic retinopathy; NPDR, non-proliferative diabetic retinopathy; PDR, proliferative diabetic retinopathy

*significancy of p-value

### Differences in retinal fractal dimension based on diabetic retinopathy severity

The Wald Test value generated FD of the SCP model yielded no significant difference among the groups (p-value > 0.05). On the other hand, the Wald Test value generated FD of the DCP model revealed a significant difference among the groups. It showed a significant difference in FD of the DCP between the moderate NPDR, severe NPDR, and PDR groups compared to the No-DR group. The coefficient value for moderate NPDR was negative, at -0.0131, indicating that FD of the DCP value of the moderate NPDR group was 0.0131 lower compared to No-DR. The coefficient value for severe NPDR was negative, at -0.0316, indicating that FD of the DCP value of severe NPDR group was 0.0316 lower compared to No-DR. Finally, the coefficient value for PDR was negative, at 0.0326, indicating that FD of the DCP value of PDR group was 0.0326 lower compared to No-DR (Table 2).

## Discussion

This study revealed significant differences in retinal VT and FD of the DCP among diabetic patients based on DR severity. A study by Vujosevic et al. found changes in the SCP and DCP in type 1 DM patients without clinical DR, whereas in type 2 DM, the DCP was affected first. Similarly, Kim et al. reported that ischemic conditions altered choroidal vascularization in DM patients before DR signs appeared, leading to earlier microvascular changes in the DCP [19, 20]. While our main findings regarding the differences in VT and FD across DR severity stages remain consistent after accounting for inter-eye correlation, we also performed a small sub-analysis on inter-eye asymmetry, and we found no difference between the two eyes, compared to the recent study by Stino et al., that specifically investigated intereye differences in OCTA metrics in diabetic patients with the same DR stage in both eyes. Their finding of significant intereye differences (e.g., lower VD and FD in the SCP of the left eye compared to the right) is indeed very interesting and highlights that even in systemic diseases affecting both eyes, there can be subtle asymmetries [21]. 

In our study, we applied simple tortuosity measurement methods, by calculating the ratio of the actual blood vessel distance to the straight line connecting the ends of the blood vessels seen in the OCT-A image. This method is quite often used in studies of microvascular changes in DR patients, kidney disorders, sickle cell retinopathy to ROP. Almost the same as the previous study using a 3 × 3 mm OCT-A scan, this study used a 6 × 6 mm OCT-A scan and took a representation of four blood vessels per OCT-A scan. Another method that has also been done using fundus photos that are processed manually or semi-automatically, and can distinguish between arteries and veins. However, from the results of several studies, there was no significant difference between the tortuosity values of arteries and veins in patients with diabetes mellitus [14, 22]. 

Our study showed a significant increase in retinal VT with worsening DR severity. The onset and progression of VT, whether arterial, venous, capillary, or mixed in nature, are influenced by a number of factors, including hypoxia, variations in shear stress, instability in mechanical pressure, and vascular remodelling processes, which include the proliferation of endothelial and smooth muscle cells and the breakdown of the elastic lamina. Often, these systems work in tandem. Elevated VEGF levels, alongside inflammatory mediators such as interleukin-6 (IL-6) and interleukin-8 (IL-8), contribute to endothelial dysfunction, inducing fenestration, fragmentation, disruption of tight junctions, basement membrane damage, and aberrant neovascularization. VEGF also activates tip cells, which extend filopodia to direct sprouting vessel growth [23]. 

Diabetic retinopathy exemplifies these mechanisms, with increased tortuosity and neovascular changes correlating closely with disease duration and severity. Vascular tortuosity, as a marker of autoregulatory response to elevated hydrostatic pressure, has been extensively studied in DR. Fayed et al., using macular OCT-A of SCP, demonstrated a significant increase in perifoveal tortuosity with worsening DR severity. However, no substantial differences were observed between the normal eye group and the no-DR diabetic group, nor between the NPDR and PDR groups. Notably, PDR patients who had undergone panretinal photocoagulation (PRP) exhibited reduced perifoveal tortuosity compared to untreated PDR cases [14]. 

As DR gets worse, the hypoxic burden rises. This causes the vascular tree to change structurally in PDR, including becoming more tortuos. After receiving enough PRP, the hypoxic drive decreases, which results in a more normalized look of retinal vessels, including less tortuosity [24]. FD showed a similar trend to VT, with eyes with PDR treated with PRP showing a considerably larger fractal dimension than eyes without PDR. However, FD remained significantly reduced after PRP, in contrast to tortuosity, which did not significantly differ from healthy controls. This suggests that following PRP, VT normalizes more than FD, suggesting that perifoveal tortuosity may be a more useful and sensitive measure of treatment response. According to these results, VT might be a useful biomarker for evaluating PRP’s effectiveness in upcoming long-term research [14]. 

Both our study and Fayed et al.,‘s focused on perifoveal VT due to its impact on foveal integrity and central vision. These findings align with peripapillary tortuosity studies using fundus photography, which reported significantly higher arteriolar tortuosity in NPDR eyes compared to no-DR and normal eyes [8, 14, 25]. However, inconsistencies exist across studies, likely due to variations in imaging protocols and processing methods, limiting the practical application of VT as a diagnostic or monitoring tool. Still, VT remains a promising early biomarker of microvascular changes preceding clinical DR [26]. 

Regarding the observed decrease in VT in severe stages of DR, particularly in the PDR group where PRP is commonly performed, its interpretation warrants careful consideration. While an increase in VT is typically associated with earlier DR progression due to factors like altered hemodynamic forces, inflammation, and angiogenic, the subsequent decrease in advanced stages following PRP is unlikely to represent a true restoration of pristine vascular geometry. Instead, this phenomenon is more plausibly attributed to a combination of altered hemodynamic flow patterns and adaptive vascular remodelling [22, 24]. 

Our result revealed that FD of the DCP was significantly reduced in eyes with moderate NPDR, severe NPDR, and PDR compared to eyes without DR, consistent with progressive microvascular dropout and architectural simplification. However, a statistically significant difference in FD was not observed between the non-DR group and the mild NPDR group. This finding suggests a potential threshold effect, where the extent of microvascular damage and capillary non-perfusion in mild NPDR may not be extensive enough to induce a statistically significant alteration in the overall fractal architecture of the DCP. While subtle changes in individual capillaries or localized microaneurysm formation might occur at this early stage, the global complexity of the vascular network, as measured by FD, appears to be relatively preserved until the disease advances to moderate NPDR where more widespread capillary loss and remodelling become evident. While FD is a robust marker for established DR progression, very early changes in mild NPDR might be better captured by other biomarkers or techniques. For instance, more localized analyses or other quantitative measures might offer higher sensitivity in detecting the earliest microvascular alterations. Our findings align with previous studies by Bhardwaj et al. and Vujosevic et al., showing that as DR worsens, alterations brought on by diabetes mellitus initially affect DCP and then SCP. This could be a result of DCP’s lower vascular density than SCP, which leaves it more vulnerable to hypoxia-induced harm. The SCP fractal dimension analysis results in this study were minor, which could be explained by some of the aforementioned findings [17, 19]. 

Our prior study linked renal impairment, DR, arterial stiffness, and retinal neurovascular parameters, suggesting that OCT/OCT-A metrics could detect early microvascular changes reflecting systemic vascular alterations in T2DM [11]. Early detection is critical for preventing disease progression. From this study, it can be concluded that OCT-A images can be considered as a screening tool in DM patients before the appearance of clinical signs of DR and there are still many opportunities to explore other vascular geometry parameters for cases of DR and diseases that can affect other retinal vessels.

This study has several strengths, including its statistically significant findings of the VT and FD of the DCP. The results imply that early microvascular alterations may be connected to inflammation, arterial stiffness or any possible pathways that reflected in the increased VT and changed FD of retinal arteries, which may indicate microvascular dysfunction and larger cardiovascular risks. This study further supports the possibility of retinal vascular geometry parameters to enhance current screening methods as early biomarker for DR and cardiovascular disease detection, stratification and early intervention. We acknowledge that vessel density is a valuable and well-established measurable parameter in DR assessment, readily obtained with many OCTA devices. While our study focused on FD and VT, these parameters offer unique insights beyond simple vessel loss and complementary information into the geometric complexity and structural alterations of the retinal microvasculature that may complement vessel density measurements. FD, in particular, can reflect the branching pattern and space-filling capacity of the vascular network, while VT quantifies the bending and curvature of vessels, both of which are altered in DR. Although the need for external software like ImageJ for FD and VT analysis might not be ideal for rapid screening, our findings contribute to a deeper understanding of the microvascular changes in DR and suggest that these parameters, particularly in the DCP, may serve as sensitive markers of disease progression. Future advancements in automated image analysis could potentially streamline the measurement of FD and VT for clinical applications.

However, limitations include a small and imbalance sample size, absence of a normal control group and lack of analysis on systemic factors affecting VT. Due to the retrospective nature of our study and the use of archived images, a formal double-blinded assessment by multiple observers with inter-rater reliability calculation was not performed. Despite the exclusion of images with motion artifacts, some projection artifacts remained visible after automated segmentation. We believe these artifacts likely compromised the accuracy of our fractal calculations, potentially reducing the precision of our analysis and also potential bias that might arise in manual VT measurement. Future research should refine imaging and measurement methods, expand and balance the sample sizes, include control groups, and explore vascular geometry differences in DR with and without DME to standardize evaluation techniques.

## Conclusions

In comparison to the No-DR group, this study discovered a substantial rise in VT in patients with mild, moderate, and severe NPDR as well as PDR. This suggests that microvascular changes advance with the severity of DR. Furthermore, a significant decrease in FD of the DCP was found in moderate NPDR, severe NPDR, and PDR. These findings underscore the necessity of evaluating specific retinal layers to gain deeper insights into the vascular changes linked to DR. If validated in larger studies, retinal vascular geometry could become a dual-purpose screening tool for both diabetic eye disease and systemic cardiovascular risk assessment.

## Data Availability

No datasets were generated or analysed during the current study.

## Abbreviations

BCVA: Best Corrected Visual Acuity
DCP: Deep capillary plexus
DIORS: Diabetic Ocular Renal Surabaya Study
DM: Diabetes mellitus
DR: Diabetic retinopathy
FD: Fractal Dimension
FFA: Fundus fluorescein angiography
HbA1c: Haemoglobin A1c
IL-6: Interleukin 6
IL-8: Interleukin 8
IRMA: Intraretinal microvascular anomalies
NLR: Neutrophil-leukocyte ratio
NPDR: Nonproliferative diabetic retinopathy
OCT: Optical coherence tomography
OCT-A: Optical coherence tomography angiography
PDR: Proliferative diabetic retinopathy
RPE: Retinal pigment epithelium
PRP: Panretinal photocoagulation
ROP: Retinopathy of prematurity
SCP: Superficial capillary plexus
SD-OCT: Spectral-domain optical coherence tomography
T2DM: Type 2 diabetes mellitus
VT: Vessel tortuosity
